# The Sexual OBjectification and EMotion database: A free stimulus set and norming data of sexually objectified and non-objectified female targets expressing multiple emotions

**DOI:** 10.3758/s13428-021-01640-3

**Published:** 2021-07-21

**Authors:** Daniela Ruzzante, Bianca Monachesi, Noemi Orabona, Jeroen Vaes

**Affiliations:** Department of Psychology and Cognitive Science, University of Trento, Corso Bettini 81, 38068, Rovereto, Trento, Italy

**Keywords:** Sexual Objectification, Emotions, Database, Validated Stimuli

## Abstract

Sexual objectification – perceiving or treating a woman as a sexual object – is a widespread phenomenon. Studies on sexual objectification and its consequences have grown dramatically over the last decades covering multiple and diverse areas of research. However, research studying sexual objectification might have limited internal and external validity due to the lack of a controlled and standardized picture database. Moreover, there is a need to extend this research to other fields including the study of emotions. Therefore, in this paper we introduce the SOBEM Database, a free tool consisting of 280 high-resolution pictures depicting objectified and non-objectified female models expressing a neutral face and three different emotions (happiness, anger, and sadness) with different intensity. We report the validation of this dataset by analyzing results of 134 participants judging pictures on the six basic emotions and on a range of social judgments related to sexual objectification. Results showed how the SOBEM can constitute an appropriate instrument to study both sexual objectification per se and its relation with emotions. This database could therefore become an important instrument able to improve the experimental control in future studies on sexual objectification and to create new links with different fields of research.

## Introduction

Sexual objectification is a pervasive phenomenon, in which the negative consequences impact the everyday lives of women. When a woman is objectified, she is considered only for her appearance and bodily functions, often seen as an instrument without regard for her personality and dignity (Fredrickson & Roberts, 1997). Studies on sexual objectification and its consequences are numerous and cover multiple areas of research ranging from its clinical ramifications to the study of mind perception and its cognitive and neural underpinnings. Even though the research on sexual objectification has been growing steadily in the last decade, no standardized and pretested pictorial stimuli have been made freely available. The current article contains the normative data of a rich set of objectified and non-objectified female stimuli that should allow researchers to improve the experimental control in their experiments, facilitate comparisons across studies, and facilitate exact replications of their results. Moreover, the current database includes pictures of different models expressing a variety of emotions allowing the research on sexual objectification to expand and widen its link to related fields of research.

### Sexual objectification

Objectification occurs whenever someone becomes something. In the case of sexual objectification, this someone is typically a woman whose body or body parts are seen as mere instruments, separated from her personality and individuality, regarded as if they were capable of representing her (Fredrickson & Roberts, 1997). Although there is evidence that men can also be sexually objectified (e.g., Rohlinger, 2002), the phenomenon is strongly connected to the female body and most of the existing literature focused on the sexual objectification of women. Up to now, the literature has focused on defining the main consequences of this pervasive phenomenon. A plethora of studies showed how sexual objectification implies the denial of humanity and human characteristics (Heflick et al., 2011; Loughnan et al., 2010; Vaes et al., 2011; see Puvia & Vaes, 2013). As a consequence, objectified women become more likely victims of sexual violence and increase the acceptance of rape myths (Burgess & Burpo, 2012; Wright & Tokunaga, 2016). However, sexual objectification not only influences the way in which women are perceived and evaluated, they are often represented as such in the media (Fredrickson & Roberts, 1997; Goffman, 1979; Kilbourne & Jhally, 2000) and treated in objectifying ways in real life. Indeed, women reported to be victims of sexism and objectifying events more than men did (Swim et al., 2001). Specifically, recent studies showed that this happened once every 2 days (Holland et al., 2017; Koval et al., 2019).

Yet, another important consequence of sexual objectification is the phenomenon of self-objectification. Living in an objectified culture leads women to internalize an observer’s perspective, viewing their own body as an object to be looked at and evaluated only on the basis of its appearance. This phenomenon is especially problematic knowing that it has been related to multiple mental health issues, such as depression or eating disorders (Calogero & Thompson, 2009; Fredrickson et al., 1998; Gay & Castano, 2010; Jones & Griffiths, 2015; Peat & Muehlenkamp, 2011; Quinn et al., 2006; Steer & Tiggemann, 2008; Tiggemann & Williams, 2012). Apart from its clinical ramifications, sexual objectification has been investigated from a cognitive and neural perspective. Specifically, researchers have tried to study how people perceive objectified women and what neural mechanisms are involved when an observer objectifies a woman. For example, Vaes et al. (2019), looking at participants’ neural activity, showed how objectified women were perceived and elaborated as more similar to objects compared to other human beings. Relatedly, Bernard et al. (2012) applied the inversion effect to objectified male and female stimuli and showed that participants recognized inverted female targets better than inverted male targets. This result was the first one to demonstrate how a sexualized woman is elaborated more analytically dividing her body in separate body parts as if she was an object. Perceiving objectified women as object-like rather than as a full human being has been linked to a shift in focus from the face to the female body, a process that was recently confirmed by Andrighetto et al. (2019). These researchers implemented the change blindness paradigm showing how changes in the bodies of sexualized targets were more easily detected than changes in the bodies of non-sexualized targets, a difference that did not occur when the changes needed to be detected in the faces of these targets.

### Emotion recognition

Evidence that observers pay less attention to the face when facing an objectified woman raises important considerations on how the perception of others and interpersonal interactions unfold in these specific contexts. Recent studies reported that altering or manipulating the typical holistic configuration of the face (two eyes above a nose, above a mouth) decreases humanizing perception of individuals (Fincher et al., 2017; Wilson et al., 2018). Similarly, the attribution of mental states to others may influence the perception of visual social stimuli, like faces (Teufel et al., 2010). In line with these bidirectional influences between face perception and (de)humanizing processes, it is not surprising that a shift in focus from the face to a sexualized body results in the denial of human characteristics to objectified women (e.g., Heflick et al., 2011; Loughnan et al., 2010; Vaes et al., 2011).

The face, however, not only represents a static configuration distinguishing human from non-humans: the face also dynamically produces signals critical for non-verbal communications during social interactions. More specifically, humans’ social life relies on the ability of perceiving and decoding individuals’ affective information in order to adaptively understand, prevent, and respond to their mental states, intentions, and behaviors (Mitchell & Phillips, 2015). The human face is the main visual-biological stimulus from which the observer rapidly infers this socio-affective information about interlocutors (Palermo & Rhodes, 2007). Indeed, supported by a complex and highly distributed neural system (Haxby & Gobbini, 2011), face processing allows individuals to identify a person (e.g., gender, ethnicity, age, and eventually the person’s name), and to infer her/his intentions and internal affective states through facial expressions (Graham & LaBar, 2012). A pioneering theoretical framework on emotion (Ekman, 1999) refers to facial expressions as the intrinsic connections between the internal emotional state and its direct external manifestation. As such, the emotional expressive face may be considered as the bridge to understand people’s mind (Chakrabarti & Baron-Cohen, 2006; Frith & Frith, 2012). Even though the exact relationship between the processes of emotion perception and mind perception is still unclear (Mitchell & Phillips, 2015), they are clearly closely connected.

Therefore, given the critical role of the face in vehiculating individuals’ emotional states, it seems crucial to extend the research on sexual objectification including the role of emotions in understanding the attribution of a mind to objectified women. Facial expressions represent a meeting point between affective and social dynamics, they have been employed as stimuli in numerous and connected research fields dealing with cognitive, social, and emotional processes, as well as emotional disorders and psychopathology. Interestingly, these same scientific fields have been studied within the realm of sexual objectification, that is, cognitive objectification (Andrighetto et al., 2019; Bernard et al., 2018; Vaes et al., 2019), the socialization and competition between women (Vaillancourt & Sharma, 2011), emotional mechanisms (e.g., empathy, Cogoni et al., 2018) and the negative psychological consequences of self-objectification related to emotional disorders and psychopathology (e.g., depression, social anxiety, eating disorders, Jones & Griffiths, 2015; Fredrickson et al., 1998). Taken together, it is evident how sexual objectification is a widespread phenomenon that can be linked to many other research areas: from its clinical ramifications, its links with mind and face perception studies, the analysis of its underlying cognitive mechanisms, to the area of emotion perception and recognition. To allow the study on sexual objectification to expand further, the current article proposes a rich set of standardized pictorial stimuli that offers the possibility to manipulate and integrate both the objectification (or not) of female targets as well as their emotional expression.

### Existing stimuli

Studies about face perception and emotions typically have different standardized face databases at their disposal, such as the Karolinska Directed Emotional Faces (KDEF; Lundqvist et al., 1998), the MPI Facial Expression Database (Troje & Bülthoff, 1996), or the Chicago Face Database (CFD; Ma et al., 2015), to name but a few. These databases usually consist of pictures of faces with different emotional expressions all controlled for a range of important dimensions, like luminance, size, appearance, race prototypicality, etc. As a result, their usage allowed to increase the internal validity of the experimental designs facilitating the replication of studies. Research in the realm of sexual objectification, instead, did not have access to a standardized set of stimuli able to measure and manipulate variables in a controlled and objective way. Up to now, most researchers typically collected a series of pictures taken from the internet in order to depict women in a sexualized or objectified manner. For example, Murnen et al. (2003) trying to study how media images promote a thin, sexy ideal of women, selected pictures of famous women wearing revealing clothes. In all of these pictures, women were wearing different clothes, and their entire body was shown except for the feet. In contrast, Aubrey et al. (2009) used highly objectified pictures taken from women’s magazines. Moreover, in order to create the non-objectified condition, they “painted” clothes on the body of each model to cover up their sexualized body part. Following yet another approach, Vaes et al. (2011) captured the concept of objectification selecting a large number of pictures from advertisements controlling for face-ism (i.e., the ratio between the amount of face and the rest of the body that is shown in a picture, Archer et al., 1983) and the extent to which the person in each picture was judged to be objectified.

Other researchers, instead of taking pictures from the Internet, photographed sexualized and non-sexualized men and women using different types of manipulations. For example, Gervais et al. (2012) and Gervais et al. (2013) showed how women’s bodies are reduced to their sexual body parts presenting photos of white college-aged men and women from head to knee wearing a white tank top and dark long pants. They also Photoshopped the female targets’ bodies to manipulate the extent to which they approached an ideal body shape. Similarly, in a series of studies, Bernard et al. (2015) and Bernard et al. (2018) used upright and inverted pictures of men and women that were photographed both in a non-sexualized and in a sexualized condition, while Bernard et al. (2019) used similar photos of sexualized and non-sexualized men and women that were photographed controlling for their postures. Importantly, none of these sets of stimuli were made easily available to other researchers or were normed and pretested for purposes other than those under investigation.

As a result, all studies that have been conducted have used a different approach to select their stimuli, resulting in small but potentially significant differences in definitions of what objectification is, reducing both the internal and external validity of this line of research. Even though most of the studies cited above tried to exclude important confounds between pictures by controlling the targets’ posture, clothes or facial expression, and usually pre-testing the selected pictures on the basis of different social judgements (e.g., the level of attractiveness, objectification, etc.), the lack of a single standardized set of stimuli has already led to important discussions within this line of research. A case in point is the first work on the sexualized body inversion hypothesis introduced by Bernard et al. (2012). This study has been questioned due to the use of non-standardized and controlled target stimuli. Indeed, the original stimuli seemed to differ in the amount of asymmetry in body postures not only between male and female targets, but also between the inverted and upright female targets influencing the outcome of the experiment in the expected direction (Schmidt & Kistemaker, 2015; Tarr, 2013; Zogmaister et al., 2020). Even though the sexualized body inversion effect has been replicated with other stimuli, these debates could be avoided introducing a set of standardized pictorial stimuli.

All in all, the use of different pictures that reflect slightly different definitions of sexual objectification in each study together with differences in the amount of experimental control of the pictures, makes it very hard to make reliable comparisons between studies. Therefore, we deem it has become fundamental for this line of research to give researchers the possibility to select their experimental stimuli from a set of standardized and validated pictures in order to increase the experimental control within studies, to allow replications and comparisons across studies, and to extend the research within this field including emotions.

## The current database

In the current article, we present the Sexual Objectification and Emotion Database (SOBEM), a set of 280 pictures able to manipulate female sexual objectification together with the emotional expression of both objectified and non-objectified female targets. Objectification was manipulated by controlling the level of skin exposure (underwear vs. sweater). Previous research has shown how both the targets’ posture and revealing clothing can independently change the extent to which they are objectified (Bernard et al., 2019; Murnen et al., 2003; Vaes et al., 2011, 2019). However, much of the scientific evidence on sexual objectification and its link with other factors, such as the denials of humanity, comes from studies where female targets presented in bikini or underwear are perceived as less human and are more objectified than fully dressed female targets (Cogoni et al., 2018; Heflick et al., 2011; Loughnan et al., 2010; Vaes et al., 2011). Since our first aim was to manipulate both objectification and emotions, we opted to manipulate the clothing of each model keeping their aspect (absence of any make-up) and posture as neutral as possible. Two further practical reasons determined our choice: on the one hand, it is harder to control and standardize the models’ posture between pictures. On the other hand, the manipulation of the body’s posture of the female targets might distract and interact with participants’ capacity to correctly recognize the emotions that were expressed. For a similar reason, we decided to control the models’ hairstyles. Models were photographed both with their hair loose and with their hair tight into a tail. In this way, researchers who are interested in testing research questions in which emotion recognition is important might prefer models with their hair tight into a tail, while studies focusing on the effects of sexual objectification might prefer models with loose hair.

Regarding the emotions, each model expressed anger, happiness, and sadness with a low and high intensity, allowing the current database to be used for creating morphed images that gradually go from a neutral face to the expression of a full-blown emotion. Moreover, the presence of pictures in which the target expresses a specific emotion with low intensity allows for the creation of ambiguous stimuli which might be interesting to test certain hypotheses (see Discussion).

We tested whether this database could effectively manipulate both the perceived objectification of the female target and the emotion she expressed by asking participants to judge and rate each picture. To verify the correct recognition of the expressed emotions, all the pictures were evaluated on the basis of the six basic emotions (anger, disgust, sadness, fear, happiness, and surprise). In addition, also the neutrality of the facial expression was judged for each picture. Therefore, we expected the pictures expressing an emotion with high intensity to be rated as expressing that specific emotion (i.e., anger, sadness, or happiness) more than the other basic emotions, while the low-intensity emotional expressions were expected to be evaluated as more ambiguous.

In addition, we also wanted to confirm that the differences in clothing of the female targets (underwear vs. sweater) could significantly shift the perceived sexual objectification of the female targets. Therefore, the target in each picture was judged on the extent to which she was objectified. In line with previous research (Loughnan et al., 2010; Vaes et al., 2011; Vaes et al., 2019), we expected the scarcely dressed female targets to be objectified significantly more than the fully dressed targets. Furthermore, we investigated some of the social judgments that have shown to be central in the literature on sexual objectification or to be correlated with this phenomenon (i.e., attractiveness, sexiness, competence, likeability, and trustworthiness). First of all, we wanted to control the level of attractiveness attributed to each model in each picture. Given that all the pictures depicted highly attractive and professional models, we did not expect any significant differences regarding the evaluation of attractiveness for objectified and non-objectified targets. The level of sexiness has shown to be correlated with the level of objectification of the female targets in past research (e.g., Fasoli et al., 2018; Vaes et al., 2011). In line with this finding, we expected objectified female targets to be perceived and evaluated as sexier than their non-objectified counterparts. On the basis of the results of Heflick et al. (2011), we wanted to show that the objectified models are perceived to be less competent and trustworthy. Finally, we also measured the likeability of each target. In the literature, contrasting results have emerged about the perceived likeability of objectified and non-objectified female targets. While Heflick et al. (2011) suggested that objectified women were seen as less warm and likeable than their non-objectified counterparts, Gray et al. (2011), instead, demonstrated that objectified female targets were evaluated as warmer and likeable than those who were not objectified. For this reason, we did not have any a priori hypothesis about the perceived likeability of the objectified and non-objectified female targets.

### Hypotheses

Our specific hypotheses are as follows:

- H1: We aimed to present a picture dataset that could manipulate different emotional expressions; therefore we expected all three emotions (i.e., anger, sadness, and happiness) to be recognized coherently. Moreover, we expected the high-intensity emotional expressions to be recognized more correctly, while the low-intensity emotional expressions were expected to be evaluated more ambiguously.
- H2: We also aimed to present a picture dataset that could successfully manipulate sexual objectification. For this reason, we expected the scarcely dressed female targets to be objectified significantly more than the fully dressed targets. The literature on sexual objectification has consistently shown that sexualized women are objectified by both heterosexual men and women (Cogoni et al., 2018; Heflick et al., 2011; Vaes et al., 2019, 2020). For this reason, we expected female and male participants to evaluate objectified and non-objectified models in a similar way.
- H3: In addition, and in line with previous research, we also hypothesized that objectified female models were perceived as sexier, less competent and less trustworthy than the non-objectified ones. We did not have any a priori hypotheses about the perceived attractiveness and likeability of the objectified and the non-objectified targets, but we measured these dimensions, so that, researchers could control their selected pictures on these dimensions.

We did not have specific hypotheses regarding the way in which the models’ hair was presented, but we verified whether the hairdo of the models (loose vs. tight into a tail) might influence their social evaluation.

## Method

### Collecting stimuli

Through a professional modeling agency, we invited ten Caucasian models into the laboratory. Upon arrival, each model was asked to read and sign a consent/release form, allowing us to use their photos for research purposes only. Afterwards, they were asked to wear a neutral black jumper first (fully dressed, non-objectified condition) and then a black brassiere that we gave them (scarcely dressed, objectified condition). The first set of photos of each dress condition was taken asking the models to let their hair down and then to pull their hair back into a ponytail. They were seated at a fixed distance from a digital camera that we adjusted to the model’s eye level following the rule-of-thirds grid. Behind them, a white panel was adjusted in order to have the same uniform background for everyone. Two photo lamps were placed on each side in front of the model in order to control the lighting conditions and to standardize shadows for everyone. All the pictures depict the models while expressing a neutral expression and the three different emotions: happiness, sadness, and anger, with different intensity in the four different conditions (objectified condition with hair loose and hair tight into a tail and non-objectified condition with hair loose and tight into a tail). In order to have different intensities in the expression of the emotions, we asked each model to simply slowly make the expression while we were taking the pictures. In this way, we collected sequences of the whole expression from the beginning to the end. This resulted in multiple photographs for each model making each of the different facial expressions. Photographs were taken to include the waist and head using an 18 to 55-mm 3.5–5.6 f lens. The photographs were shot in high-resolution, raw format.

### Standardization of stimuli

**Fig. 1:**
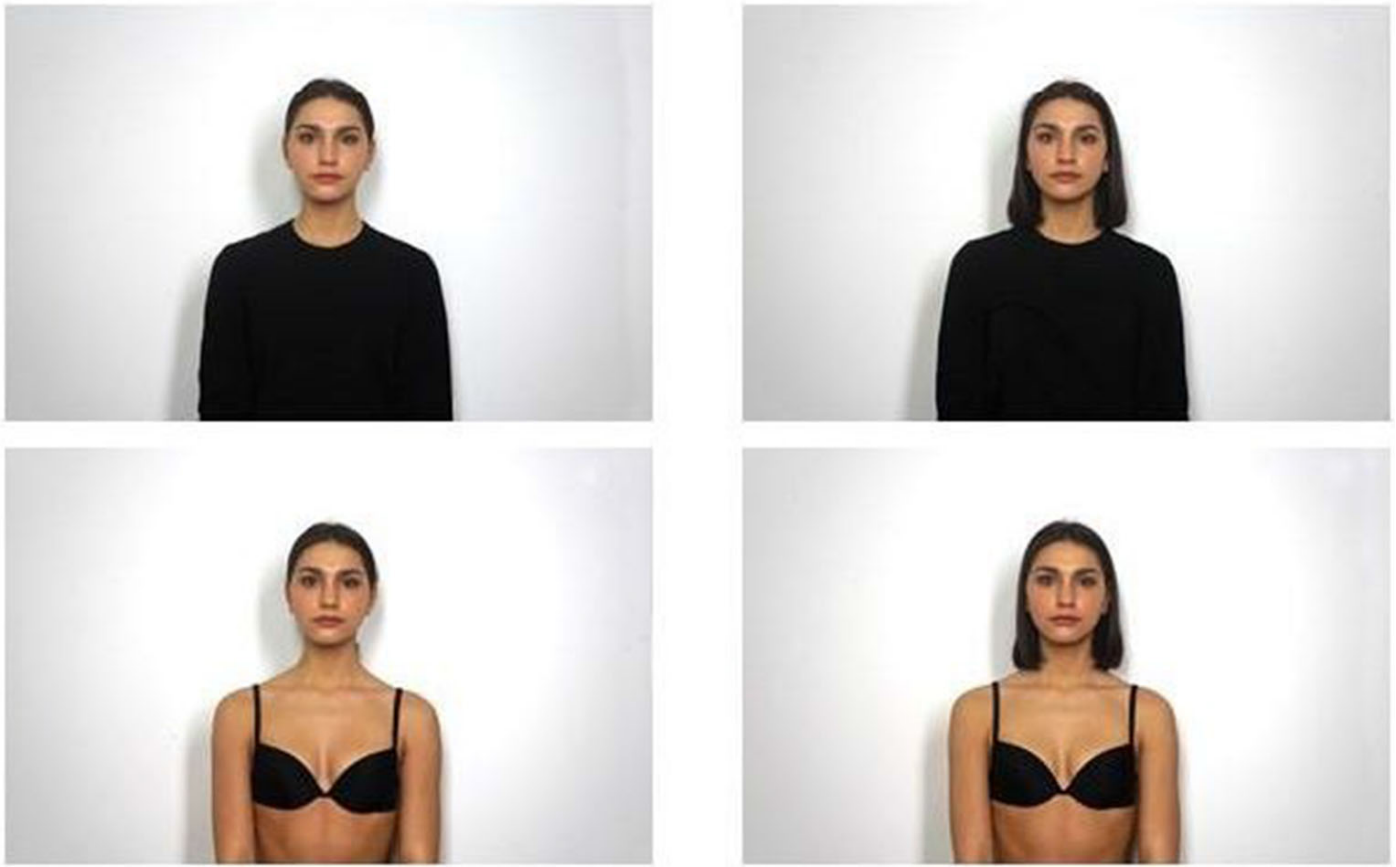
Examples of stimuli. These first four pictures depict a model with neutral expression in the four different combinations of objectification and hairstyle conditions available in the picture dataset (first line: non-objectified; second line: objectified; first column: hair tight into a tail; second column: hair loose)

**Fig. 2:**
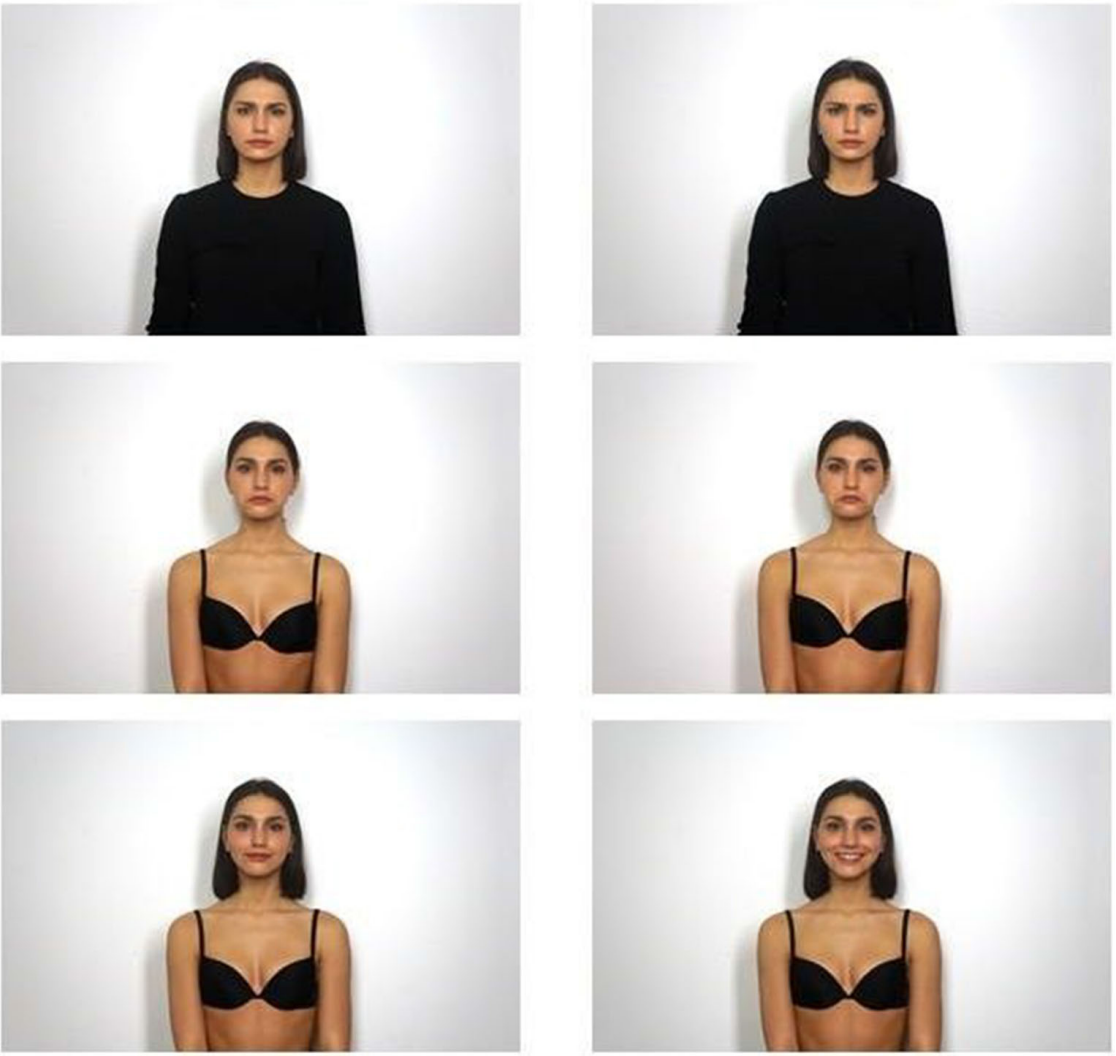
Examples of stimuli. These pictures represent the three different emotions (anger, sadness, and happiness). On the left side, emotions are presented with low intensity while on the right side with high intensity

We first selected one image of each model in each condition displaying a neutral facial expression with the head and body of the person in a straight position. Afterwards, we proceeded by selecting the emotional expression images focusing on the quality and clarity of the emotional expression. All the image files were then edited using Adobe Photoshop. We modified the pictures by removing facial and body moles, earrings, and facial piercings. We also resized the pictures. The original dimensions of the photos were 5456 pixels (wide) × 3632 pixels (high). To standardize the size of the photo, we created an invisible 5171 pixels (wide) × 3320 pixels (high) rectangle in which we created two lines: the first one was a vertical line at the middle of the rectangle, while the second one was the first horizontal line of the thirds grid. The rectangle was applied over the pictures such that the center of the rectangle met the chin while the point where the two lines intersected corresponded to the nose of the person. Finally, images were equated for color temperature by setting a white point near the face of each model. In this way, a total of 280 pictures were created showing one of seven facial expressions [Neutral (NE), Anger low-intensity (AN1), Anger high-intensity (AN2), Sadness low-intensity (SA1), Sadness high-intensity (SA2), Happiness low-intensity (HA1), Happiness high-intensity (HA2)], either fully- (non-objectified condition) or scarcely dressed (objectified condition), with their hair tight into a tail or loose (see Figs. 1 and 2 for stimuli example).

### Participants

A total of 139 participants were involved in an online experiment conducted on Prolific Academic where they received a fee of 3.75 £ for their participation. A total of 20 attention checks were presented in a randomized order in each questionnaire. We created two different types of attention checks: one in which we simply indicated participants to write a specific word in a textbox presented below, while a second attention check asked participants to select a specific number on a Likert-scale. Participants who failed more than one attention check were excluded from the analysis. A final sample of 134 participants (71 male, *M*_*age*_ = 24.3, *SD* = 3.9) were retained for the analysis; 112 of them were white or Caucasian, five black or from African descendent, 13 Asian, one Japanese, and three preferred not to specify their racial identity. All participants were heterosexual, except for one who reported to be bisexual.

### Procedure

Four different versions of the questionnaire were created in order to make sure that each participant was presented with all ten models and that all four conditions appeared in each version. Specifically, in each version, every single model expressed all of the possible emotions (neutral, sad-high, sad-low, happy-high, happy-low, anger-high, and anger-low), but in only one of the possible combinations between dress and hairstyle (e.g., in version 1, model 1 appeared with her hair tight into a tail and in bikini, in version 2 the same model was presented with her hair tight into a tail and fully dressed, in version 3 with her hair loose and in bikini, and in version 4 with her hair loose and fully dressed). The combinations between dress and hairstyle changed in each version across models. As such, in each questionnaire, a total of 70 pictures were presented that included all ten models each showing all the facial expressions.

Questionnaires first asked participants to sign the informed consent form followed by reporting participants’ demographics such as age, gender, sexual orientation, and racial identity. Afterwards, each picture was presented at the top of the page under which a series of questions appeared. Participants were asked to evaluate to what extent the person in the picture had a neutral expression and then had to indicate the intensity with which each picture expressed each of the six basic emotions (i.e., anger, fear, disgust, happiness, sadness, and surprise) on a seven-point Likert type scale (1 = *Very weakly* to 7 = *Very strongly*). Following this task, they were asked to evaluate each model on the basis of their attractiveness, competence, trustworthiness, sexiness, likeability, and finally they had to express the extent to which the woman in the picture was objectified. All these judgments were made on a seven-point Likert type scale (1 = *Not at all* to 7 = *Extremely*).

### Analytical strategies

We used the pictures as a unit of analysis aggregating the judgements across participants separately for male and female participants. As such, each judgment was based on the evaluation of 15 to 17 participants. Therefore, three different power sensitivity analyses were performed separately for each analysis.

### Identification of facial expressions

In order to verify whether the emotional expressions on average were correctly identified by participants, a repeated measures ANOVA was conducted in a 7 (Judgement: Neutral expression, Angry, Disgust, Fear, Happiness, Sadness, Surprise) X 2 (Objectification: Objectified, Non-Objectified) X 2 (Hair: Loose, Tight into a tail ) X2 (Gender: Women, Men) experimental design, with the Judgment as within-participants factor and the Objectification, Hair and Gender as between-participants factors. This analysis was conducted for each of the seven emotion expressions separately (i.e., neutral, low-, and high anger, sadness and happiness).

A power sensitivity analysis was performed for the emotional analysis using PANGEA (for details see www.jakewestfall.org/pangea/). The sensitivity power analysis indicated that with 80 observations we had sufficient power (.806) to detect an effect size of *d* =.25 with *η*_*p*_^*2*^ = .016 and with an alpha = 0.05 for the main effect of emotional judgment. Therefore, our current experimental setup allowed us to reliably detect medium effects for the emotional analyses. To specifically validate the effectiveness of the high- and low-intensity manipulation of facial expressions, a univariate analysis of variance was performed *separately* on each relevant emotional rating (i.e., Angry, Sad, and Happy) comparing the judgments of the pictures’ intensity (Low, High), Objectification (Objectified, No-Objectified), Hair (Loose, Tight into a tail) and participants’ Gender (Women, Men) as between-participants variables in a 2 x 2 x 2 x 2 experimental design. A second sensitivity power analysis was performed for this analysis using PANGEA (for details see www.jakewestfall.org/pangea/). In this case, analysis indicated that with 160 observations we had sufficient power (.801) to detect an effect size of *d* =.13 with *η*_*p*_^*2*^ = .004 and with an alpha = 0.05 for the main effect of low and high intensity. Therefore, our current experimental setup allowed us to reliably detect small effects.

### Social judgments

For the social judgment analyses, we conducted univariate ANOVAs for each social judgment separately. Each ANOVA had a 2 (Objectification: Objectified, Non-Objectified) X 2 (Hair: Loose, Tight into a tail) X 2 (Gender: Women, Men) between-participant design. In all statistical analyses, the alpha level was set to .05 and all pairwise comparisons were Bonferroni-corrected. A third sensitivity power analysis was performed for the social judgements’ analysis using PANGEA (for details see www.jakewestfall.org/pangea/). With a total of 560 observations, we had sufficient power (.806) to detect an effect size of *d* = .091 with *η*_*p*_^*2*^ = .002 and with an alpha = 0.05 for the main effect of objectification. Therefore, our current experimental setup allowed us to reliably detect small effects for this analysis.

## Results

### Identification of facial expressions

#### Evaluation of facial expressions as a function of Judgement, Objectification, Hairstyle, and Gender

For reasons of brevity, only significant main and interaction effects are reported. Means and standard deviations are reported in Table 1. All reported analyses follow the Greenhouse–Geisser correction, because Mauchly’s sphericity could not be assumed.

**Table 1:** Means and standard deviations of each emotional expression qualified by Objectification, Hair, and Gender for the most rated emotions. In addition, the general mean and standard deviation of the most rated non-target emotion is indicated

Emotional expression	Most rated emotion	Objectification		Hair		Gender		Most rated non-target emotion	Most rated non-target emotion
		Objectified	Non-objectified	Loose	Tight into a tail	Male	Female		
		*M (SD)*	*M (SD)*	*M (SD)*	*M (SD)*	*M (SD)*	*M (SD)*		*M (SD)*
**Neutral**	Neutral	5.27 (.79)	5.53 (.63)	5.40 (.81)	5.40 (.64)	5.37 (.73)	5.42 (.73)	Sadness	2.45 (.63)
**Anger Low**	Anger	3.79 (.95)	3.87 (.94)	3.98 (.98)	3.68 (.90)	3.72 (.88)	3.94 (1.01)	Disgust	3.74 (.71)
**Anger High**	Anger	4.78 (.91)	4.73 (.84)	4.78 (.85)	4.73 (.90)	4.51 (.84)	5.00 (.84)	Disgust	4.34 (.53)
**Sadness Low**	Sadness	4.55 (1.22)	3.96 (1.10)	4.16 (1.20)	4.35 (1.19)	4.10 (1.12)	4.42 (1.26)	Neutral	3.40 (1.15)
**Sadness High**	Sadness	5.25 (1.00)	5.27 (.87)	5.26 (.94)	5.26 (.94)	5.18 (.96)	5.35 (.91)	Disgust	2.90 (.55)
**Happiness Low**	Happiness	4.91 (.70)	4.86 (.68)	4.87 (.71)	4.91 (.67)	4.94 (.62)	4.83 (.75)	Neutral	3.36 (.70)
**Happiness High**	Happiness	6.07 (.45)	6.01 (.50)	6.08 (.45)	6.05 (.50)	5.98 (.48)	6.15 (.46)	Surprise	2.24 (.42)

##### Anger-low

The main effect of Judgment, *F*(1.94, 139.86) = 107.74, *p* < .001, *η*_*p*_^2^ = .599, was qualified by a significant two-way interaction with Gender, *F*(1.94, 139.86) = 3.49, *p* = .034, *η*_*p*_^2^ = .046. Even though both men and women judged the models portrayed with a low-anger expression as angrier than all other emotions (*ps* < .001) apart from the neutrality and disgust judgment (*ps* > .99), men showed this effect more strongly than women did. Specifically, while disgust was not significantly distinguished from anger by both genders, women showed a slightly stronger tendency to distinguish anger from neutrality (*p* = .15; *M*_*Anger*_ = 3.94, *SD*_*Anger*_ = .15; *M*_Neutral_ = 3.12, *SD*_Neutral_ = .16), while men did not (*p* > .99; *M*_*Anger*_ = 3.72, *SD*_*Anger*_ = .15; *M*_Neutral_ = 3.49, *SD*_Neutral_ = .16).

##### Anger-high

Again, the main effect of Judgment, *F*(2.47, 177.50) = 363.88, *p* < .001, *η*_*p*_^2^ = .835, was qualified by a significant interaction with Gender, *F*(2.47, 177.50) = 9.15, *p* < .001, *η*_*p*_^2^ = .113. Pairwise comparisons showed that women successfully labeled the Anger expression (ratings for anger higher than all other ratings, *p*s < .001), whereas men judged the Anger expression as more intensely angry than all other emotions (*p*s < .001; *M* = 4.51, *SD* = .14), except for Disgust (*p* > .27; *M* = 4.21, *SD* = .08).

##### Sadness-low

The significant main effect of Judgment, *F*(1.66, 119.51) = 102.91, *p* < .001, *η*_*p*_^2^ = .588, was qualified by a significant interaction with Objectification, *F*(1.66, 119.51) = 4.59, *p* = .017, *η*_*p*_^2^ = .060, showing that models with sad-low expressions in the Objectified condition were properly judged as more intensely sad than all other emotions (all *ps* < .003). In the non-objectified condition, the ratings for sadness (*M* = 3.96, *SD* = .20) did not differ from the ratings of neutrality (*p* > .99; *M* = 3.68, *SD* = .18).

##### Sadness-high

The main effect of Judgment was significant, *F*(1.92, 138.18) = 330.09, *p* < .001, *η*_*p*_^2^ = .821, confirming that the ratings for Sadness were the highest compared to all other emotions (all *p*s < .001). The main effect of Gender was significant as well, *F*(1, 72) = 5.41, *p* < .023, *η*_*p*_^2^ = .070, revealing that, overall, men provided more intense emotional judgments than women.

##### Happiness-low

Only the main effect of Judgement was significant, *F*(1.64, 118.04) = 648.41, *p* < .001, *η*_*p*_^2^ = .900, confirming that happy expressions were properly labeled (ratings for happiness were the highest, *p*s < .001). In addition, a main effect of Gender emerged, *F*(1, 72) = 9.63, *p* = .003, *η*_*p*_^2^ = .118, revealing that, overall, men’s ratings were more intense than women’s ratings.

##### Happiness-high

A main effect of Judgment, *F*(1.82, 131.24) = 2617.72, *p* < .001, ${\eta}_p^2$ = .973, and of Gender, *F*(1, 72) = 11.68, *p* = .001, ${\eta}_p^2$ = .140, were both qualified by a significant interaction between both variables, *F*(1.82, 131.24) = 10.82, *p* < .001, ${\eta}_p^2$ = .131. Even though both genders rated models portrayed with a happy emotion as expressing more happiness than all other emotion (*ps* < .001), women did this more strongly than men (*M*_Women_ = 6.15, *SD*_Women_ = .08; *M*_Men_ = 5.98, *SD*_Men_ = .08).

##### Neutral

Finally, the main effect of Judgment, *F*(2.23, 160.51) = 363.51, *p* < .001, *η*_*p*_^2^ = .835, showed that photos depicting a neutral expression were properly perceived as expressing a more neutral face compared to all other emotions, all *p*s < .001. This same pattern held for both Objectified and Non-Objectified models, even though the interaction between Judgment and Objectification showed to be significant, *F*(2.23, 160.51) = 4.21, *p* = .013, *η*_*p*_^2^ = .055, (*M*_*Objectified*_ = 3.21, *SD*_Objectified_ = .16; *M*_Non-objectified_ = 3.41, *SD*_Non-objectified_ = .16).

#### Evaluation of facial expression intensity as a function of Judgement, Objectification, Hairstyle, and Gender

Emotion intensity differed significantly for all emotions as was shown by a significant main effect of Intensity in Sadness, *F*(1, 144) = 34.19, *p* < .001, *η*_*p*_^2^ = .192, Happiness, *F*(1, 144) = 149.38, *p* < .001, *η*_*p*_^2^ = .509, and Anger, *F*(1, 144) = 41.26, *p* < .001, *η*_*p*_^2^ = .223. In all cases, the ratings for low-intensity facial expressions (*M*_*anger*_ = 3.83, *SD*_*anger*_ = .94; M_*happiness*_ = 4.89, *SD*_*happiness*_ = .69; *M*_*sadness*_ = 4.26, *SD*_*sadness*_ = 1.19) were lower than those for high-intensity facial expressions (*M*_*anger*_ = 4.75, *SD*_*anger*_ = .87; M_*happiness*_ = 6.06, *SD*_*happiness*_ = .47; *M*_*sadness*_ = 5.26, *SD*_*sadness*_ = .93). For the Angry facial expressions, the main effect of Gender was significant as well, *F*(1, 144) = 6.31, *p* = .013, *η*_*p*_^2^ = .042, indicating that women’s ratings (*M* = 4.47, *SD* =1.06) were higher than those of men (*M* =4.11, *SD* = .94).

### Social judgements

#### Attractive

A main effect of Gender, *F*(1, 552) = 6.51, *p* = .011, *η*_*p*_^2^ = .012, and Hair, *F*(1, 552) = 9.62, *p* = .002, *η*_*p*_^2^ = .017, emerged (see Table 2). A significant two-way interaction between Gender and Hair, *F*(1, 552) = 6.37, *p* = .012, *η*_*p*_^2^ = .011, showed that only male participants evaluated the models with loose hair as significantly more attractive (*M* = 4.53, *DS* = .06) compared to those with hair tight into a tail (*M* = 4.18; *DS* = .06).

**Table 2:** Means and standard deviations of each social judgements qualified by Objectification, Hair, and Gender

Social judgements	Objectification		Hair		Gender	
	Objectified	Non-objectified	Loose	Tight into a tail	Male	Female
	*M (SD)*	*M (SD)*	*M (SD)*	*M (SD)*	*M (SD)*	*M (SD)*
Attractive	4.32 (.76)	4.23 (.72)	4.37 (.77)	4.18 (.71)	4.35 (.72)	4.20 (.76)
Sexy	4.01 (.78)	3.55 (.69)	3.89 (.80)	3.68 (.73)	4.00 (.75)	3.56 (.73)
Competent	3.93 (.60)	4.17 (.66)	4.06 (.64)	4.04 (.64)	4.17 (.51)	3.92 (.73)
Likeable	4.02 (.81)	4.07 (.87)	4.07 (.84)	4.03 (.84)	4.13 (.76)	3.97 (.91)
Trustworthy	3.86 (.65)	4.05 (.71)	3.96 (.69)	3.95 (.68)	4.03 (.58)	3.88 (.77)
Object	4.68 (.37)	2.45 (.42)	3.59 (1.15)	3.54 (1.22)	3.49 (1.12)	3.64 (1.24)

#### Sexy

A main effect of Objectification, *F*(1, 552) = 61.80, *p* < .001, *η*_*p*_^2^ = .101 and Gender, *F*(1, 552) = 55.35, *p* < .001, *η*_*p*_^2^ = .091, showed to be significant. As expected, objectified models were evaluated as sexier than non-objectified models and men judged the models in general as sexier than women did (see Table 2). Moreover, a significant main effect of Hair, *F*(1, 552) = 12.69, *p* < .001, *η*_*p*_^2^ = .022, showed that models with loose hair were considered as sexier than models with hair tight into a tail.

#### Competence

The main effect of Objectification, *F*(1, 552) = 20.28, *p* < .001, *η*_*p*_^2^ = .035, confirmed that objectified models were evaluated as less competent than non-objectified models. A main effect of Gender, *F*(1, 552) = 22.45, *p* < .001, *η*_*p*_^2^ = .039, showed also that female rather than male participants evaluated the models as less competent (see Table 2).

#### Likeable

Unlike the other social judgements, a single main effect of Gender, *F*(1, 552) = 4.87, *p* = .028, *η*_*p*_^2^ = .009, showed to be significant. Male participants appraised the models in general as more likeable compared to the female participants (see Table 2).

#### Trustworthy

Similar to the competence judgement, a significant main effect of Objectification, *F*(1, 552) = 10.06, *p* = .002, *η*_*p*_^2^ = .018, demonstrated that objectified models were considered as less trustworthy than the non-objectified models. Also, a significant main effect of Gender, *F*(1, 552) = 6.24, *p* = .013, *η*_*p*_^2^ = .011, showed female participants to evaluate models as less trustworthy than male participants (see Table 2).

#### Object

A significant main effect of Objectification, *F*(1, 552) = 4719.17, *p* < .001, *η*_*p*_^2^ = .895, and Gender, *F*(1, 552) = 23.24, *p* < .001, *η*_*p*_^2^ = .040, emerged (see Table 2). A significant interaction between Gender and Objectification, *F*(1, 552) = 15.56, *p* < .001, *η*_*p*_^2^ = .027, demonstrated that both men and women judged the objectified models as significantly more object-like compared to the non-objectified ones, but women showed this effect more strongly (*M*_*Objectified*_ = 4.82, *SD*_*Objectified*_ = .03; *M*_*Non-objectified*_ = 2.47, *SD*_*Non-Objectified*_ = .03) compared to men (*M*_*Objectified*_ = 4.54, *SD*_*Objectified*_ = .03; *M*_*Objectified*_ = 2.44, *SD*_*Non-objectified*_ = .03).

## Discussion

Given that sexual objectification is a widespread phenomenon, studying and understanding this phenomenon becomes fundamental. While the literature on sexual objectification has helped to unravel some of its causes and consequences, to allow researchers to make comparisons between studies and connect this phenomenon to several other fields of research, a common, standardized and comparable tool for future investigations becomes indispensable. As affective processes represent a lynchpin across different perspectives of research, from cognitive and social to psychopathology, we created the Sexual Objectification and Emotion database (SOBEM) in which objectified and non-objectified female models are presented expressing different facial emotions with different intensity.

The SOBEM database showed to be appropriate to manipulate sexual objectification. Results were in line with the current literature showing that the scarcely dressed models were more objectified than their fully dressed counterparts. Additionally, objectified models were perceived as significantly sexier, less competent, and less trustworthy compared to the non-objectified models, in line with previous research on sexual objectification (Heflick et al., 2011; Vaes et al., 2011). Furthermore, loose hair seemed to influence the evaluation of women in general. Models with loose hair were perceived as sexier than models with their hair tight into a tail. These social judgements were also influenced by the gender of participants. Indeed, in general, men tended to judge the models as more attractive, likeable, competent, trustworthy, and sexy. Instead, female participants tended to objectify the objectified models a bit more strongly. These effects were only a matter of degree given that both genders clearly differed their perceptions of the objectified and the non-objectified models in the expected direction.

In addition, results of the validation of the SOBEM showed that almost all emotions expressed by the models were perceived coherently with the manipulation of the specific emotion and with its two intensities. This was particularly true for neutral, and for happy and sad high-intensity emotions. Although high-intensity angry faces were appropriately recognized, these were also judged as expressing disgust by men. This kind of confusion has been reported before in several studies (Aviezer et al., 2008; Widen & Russell, 2010). Specifically, angry and disgusted facial expressions may be confused, in particular during an identification task, in which labeled categories are provided (Widen & Russell, 2004). Different aspects may contribute to the difficulty in distinguishing the two emotional faces: perceptively, angry and disgusted faces share similar action patterns around the forehead and the eyes (Susskind & Anderson, 2008), and, conceptually, it has been suggested (Nabi, 2002) that the word *disgust* may be misleading, and its meaning may be interpreted as expressing a compound of disgust and anger. That this confusion emerged for men and not for women may be in line with the literature suggesting a female advantage in facial expressions recognition (Wingenbach et al., 2018). In any case, it is important for researchers whose project aims to study anger expressions to take these possible misperceptions into consideration and select specific targets to avoid or diminish this problem.

Regarding the evaluation of photos portraying low-intensity facial expressions, in general, all emotions were still correctly perceived, although the ambiguity increased together with the misperception of more than one emotion at a time. Indeed, the intensity of anger was again equally high as the intensity of disgust in all conditions, whereas the perception of sadness, a negative low-arousal emotion, was as intense as the perception of a neutral facial expression for the non-objectified models. The expression of low-intensity happiness, instead, was correctly identified in all conditions suggesting that this emotion was less ambiguous than the other low-intensity emotions. This is probably due to the high human sensitivity to this kind of emotional expression: indeed, 20% of intensity is enough to recognize this kind of facial expression (vs. 40% or more for other facial expressions, Calvo et al., 2016). In addition, it is important to remind that happiness was the only positive emotion among the stimuli as well as among the labels of the six basic emotions. Overall, these results allow us to confirm that the SOBEM database can be suitable to manipulate both emotional expressions and sexual objectification.

Even though no research to date that we are aware of has integrated the study of sexual objectification and emotion recognition, the introduction of the SOBEM might facilitate interesting avenues for future research linking both areas of research. For example, self-objectification has shown to play an important role in predicting restrained eating and consequently eating disorders (Calogero & Thompson, 2009; Fredrickson et al., 1998; Peat & Muehlenkamp, 2011; Tiggemann & Williams, 2012). It has also been linked to symptoms of depression (Jones & Griffiths, 2015), sexual dysfunctions (Steer & Tiggemann, 2008), and interfering with women’s daily tasks impairing their cognitive performance (Fredrickson et al., 1998; Gay & Castano, 2010; Quinn et al., 2006). The symptoms described above all involve the impairment of emotion recognition of the self or other. Indeed, depressed patients fail in the recognition of happy and sad facial expressions (Mikhailova et al., 1996), while eating disorders sometimes co-occur with alexithymia, a syndrome characterized by difficulties in identifying feelings and in recognizing emotions (Nowakowski et al., 2013). Having a picture database able to manipulate both emotions and sexual objectification might be an important tool to better understand the interaction between different interrelated psychopathologies.

Another promising avenue for future research in which the study of sexual objectification and emotion recognition might be integrated is mind perception. The comprehension of facial expressions is dependent on the ability to mentalize (Frith & Frith, 2012), and on the development of Theory of Mind (ToM, Chakrabarti & Baron-Cohen, 2006), both concepts referring to the attribution and prediction of mental states, desires, and behaviors in other people. Given that sexually objectified women have been shown to be de-mentalized and dehumanized (e.g., Loughnan et al., 2010; Vaes et al., 2011), the current set of pictures in which such targets clearly express emotions and feelings could help us study a whole range of processes and how they affect both emotion recognition and processes of dehumanization. In addition, even though the exact relationship between the processes of emotion perception and mind perception is still unclear (Mitchell & Phillips, 2015), the current database could help clarify how these processes interact. A lot of research on dehumanization and mentalization processes have studied these processes using targets that are historically discriminated such as black faces or doll-like faces that clearly do not have a mind (Goff et al., 2008; Harris & Fiske, 2007; Wheatley et al., 2011). At the same time, race effects in the recognition of emotions have been reported repeatedly in the literature (Elfenbein & Ambady, 2002, 2003). Therefore, comparing the recognition of emotions with objectified or non-objectified women that differ in the way they are de-mentalized could help define the relation between mind perception and emotion recognition. Secondly, the current set of pictures could deepen our knowledge on empathic processes that have shown to be impaired towards sexually objectified women (e.g., Cogoni et al., 2018). Importantly, none of this research has used objectified and non-objectified targets that express emotions. Also, mimicry – the capacity to spontaneously simulate other people’s facial expressions and behaviors – has shown to be influenced by the amount of mind we attribute to a target (Hofree et al., 2014) and has shown to be important in emotion recognition (Goldman & Sripada, 2005). Both phenomena could be studied in the context of sexual objectification with this set of pictures.

Lastly, the presence of low-intensity pictures in the current database might be used to create dynamic stimuli through the use of morphing techniques. Such dynamic stimuli might allow researchers to test the impact of sexual objectification in more complex interactive context (Gervais et al., 2019). Moreover, ambiguous stimuli could be suitable to investigate how contextual factors and individual differences influence emotion recognition processes (Wieser & Brosch, 2012). Indeed, the categorization of facial expressions is strictly modulated by external contextual cues (Aviezer et al., 2011), and this is particularly true for ambiguous or non-emotional faces (e.g., Bublatzky et al., 2020; Russell & Fehr, 1987). Indeed, there is evidence that psychological disorders (e.g., social anxiety disorders, Maoz et al., 2016; eating disorders, Fujiwara et al., 2017), as well as implicit prejudice (Hutchings & Haddock, 2008) may bias the perception of ambiguous emotional faces. Therefore, the low-intensity emotion pictures in the current database could be used to investigate whether contextual aspects and stereotypes influence the processing of facial expressions within the phenomenon of sexual objectification.

## Limitations

Even if we believe this is the first picture database presented in the field of sexual objectification and its validation confirms its suitability, this work is not without limits.

Indeed, we did not include male pictures in the database. In different past studies, the main manipulation consisted in comparing the sexual objectification toward female and male targets. However, given that it has been widely demonstrated how women are the main victims of this phenomenon (Heflick et al., 2011; Vaes et al., 2019, 2020), we decided to include pictures of women only. In this way, sexual objectification can be manipulated comparing scarcely and fully dressed female targets. In addition, emotion recognition research typically keeps the gender of the targets constant to avoid gender effects. Nonetheless, future research could consider validating a database of sexualized and non-sexualized male models to allow comparisons between gender.

All models in the current picture database are Caucasian women. Even though sexual objectification is mostly observed in Western cultures (Loughnan et al., 2015), it has shown to target minority women as well and even more so than white women (Anderson et al., 2018). Therefore, extending this picture database including also non-Caucasian models is an important endeavor for future research.

## Conclusion

The SOBEM is a picture database that consists of objectified and non-objectified women expressing different facial expressions. This database aims to become a standardized tool to study sexual objectification allowing researchers to make between study comparisons and increase the experimental control within their studies. Finally, we hope it will be used to investigate and create new theoretical links across different research fields and especially in the realm of emotion recognition.

## Data Availability

See section “Accessing the SOBEM” above.

## References

[CR1] Anderson JR, Holland E, Heldreth C, Johnson SP (2018). Revisiting the Jezebel stereotype: The impact of target race on sexual objectification. Psychology of Women Quarterly.

[CR2] Andrighetto L, Bracco F, Chiorri C, Masini M, Passarelli M, Piccinno TF (2019). Now you see me, now you don’t: Detecting sexual objectification through a change blindness paradigm. Cognitive Processing.

[CR3] Archer D, Iritani B, Kimes DD, Barrios M (1983). Face-ism: Five studies of sex differences in facial prominence. Journal of Personality and Social Psychology.

[CR4] Aubrey JS, Henson JR, Hopper KM, Smith SE (2009). A Picture is Worth Twenty Words (About the Self): Testing the Priming Influence of Visual Sexual Objectification on Women’s Self-Objectification. Communication Research Reports.

[CR5] Aviezer H, Hassin R, Ryan J, Grady C, Susskind J, Anderson A, Moscovitch M, Bentin S (2008). Angry, Disgusted, or Afraid? Studies on the Malleability of Emotion Perception. Psychological Science.

[CR6] Aviezer H, Bentin S, Dudarev V, Hassin RR (2011). The automaticity of emotional face-context integration. Emotion.

[CR7] Bernard P, Gervais SJ, Allen J, Campomizzi S, Klein O (2012). Integrating Sexual Objectification With Object Versus Person Recognition: The Sexualized-Body-Inversion Hypothesis. Psychological Science.

[CR8] Bernard P, Gervais SJ, Allen J, Delmée A, Klein O (2015). From sex objects to human beings: Masking sexual body parts and humanization as moderators to women’s objectification. Psychology of women quarterly.

[CR9] Bernard P, Rizzo T, Hoonhorst I, Deliens G, Gervais SJ, Eberlen J (2018). The Neural Correlates of Cognitive Objectification: An ERP Study on the Body Inversion Effect Associated With Sexualized Bodies. Social Psychological and Personality Science.

[CR10] Bernard P, Hanoteau F, Gervais S, Servais L, Bertolone I, Deltenre P, Colin C (2019). Revealing Clothing Does Not Make the Object: ERP Evidences That Cognitive Objectification is Driven by Posture Suggestiveness, Not by Revealing Clothing. Personality and Social Psychology Bulletin.

[CR11] Bublatzky F, Kavcıoğlu F, Guerra P, Doll S, Junghöfer M (2020). Contextual information resolves uncertainty about ambiguous facial emotions: Behavioral and magnetoencephalographic correlates. NeuroImage.

[CR12] Burgess M, Burpo S (2012). The effect of music videos on college students' perceptions of rape. College Student Journal.

[CR13] Calogero RM, Thompson JK (2009). Sexual Self-Esteem in American and British College Women: Relations with Self-Objectification and Eating Problems. Sex Roles.

[CR14] Calvo MG, Avero P, Fernández-Martín A, Recio G (2016). Recognition thresholds for static and dynamic emotional faces. Emotion.

[CR15] Chakrabarti B, Baron-Cohen S (2006). Empathizing: Neurocognitive developmental mechanisms and individual differences. Progress in Brain Research.

[CR16] Cogoni C, Carnaghi A, Silani G (2018). Reduced empathic responses for sexually objectified women: An fMRI investigation. Cortex.

[CR17] Ekman P, Dalgleish T, Power MJ (1999). Facial expressions. *The handbook of cognition and emotion*.

[CR18] Elfenbein HA, Ambady N (2002). On the universality and cultural specificity of emotion recognition: a meta-analysis. Psychological bulletin.

[CR19] Elfenbein HA, Ambady N (2003). When familiarity breeds accuracy: Cultural exposure and facial emotion recognition. Journal of Personality and Social Psychology.

[CR20] Fasoli F, Durante F, Mari S, Zogmaister C, Volpato C (2018). Shades of Sexualization: When Sexualization Becomes Sexual Objectification. Sex Roles.

[CR21] Fincher KM, Tetlock PE, Morris MW (2017). Interfacing With Faces: Perceptual Humanization and Dehumanization. Current Directions in Psychological Science.

[CR22] Fredrickson BL, Roberts T-A (1997). Objectification Theory: Toward Understanding Women’s Lived Experiences and Mental Health Risks. Psychology of Women Quarterly.

[CR23] Fredrickson BL, Roberts T-A, Noll SM, Quinn DM, Twenge JM (1998). ‘That swimsuit becomes you: Sex differences in self-objectification, restrained eating, and math performance’: Correction to Fredrickson et al. (1998). Journal of Personality and Social Psychology.

[CR24] Frith CD, Frith U (2012). Mechanisms of social cognition. Annual Review of Psychology.

[CR25] Fujiwara E, Kube VL, Rochman D, Macrae-Korobkov AK, Peynenburg V (2017). Visual attention to ambiguous emotional faces in eating disorders: role of alexithymia. European Eating Disorders Review.

[CR26] Gay RK, Castano E (2010). My body or my mind: The impact of state and trait objectification on women’s cognitive resources. European Journal of Social Psychology.

[CR27] Gervais SJ, Vescio TK, Förster J, Maass A, Suitner C (2012). Seeing women as objects: The sexual body part recognition bias. European Journal of Social Psychology.

[CR28] Gervais SJ, Holland AM, Dodd MD (2013). My eyes are up here: The nature of the objectifying gaze toward women. Sex Roles.

[CR29] Gervais SJ, Allen J, Riemer AR, Gullickson M (2019). The balanced objectification hypothesis: The effects of objectification valence and body sentiment on source sentiment. Personality and Social Psychology Bulletin.

[CR30] Goff PA, Eberhardt JL, Williams MJ, Jackson MC (2008). Not yet human: Implicit knowledge, historical dehumanization, and contemporary consequences. Journal of Personality and Social Psychology.

[CR31] Goffman, E. (1979). *Gender Advertisements*. New York: Harper & Row.

[CR32] Goldman AI, Sripada CS (2005). Simulationist models of face-based emotion recognition. Cognition.

[CR33] Graham R, LaBar KS (2012). Neurocognitive mechanisms of gaze-expression interactions in face processing and social attention. Neuropsychologia.

[CR34] Gray K, Knobe J, Sheskin M, Bloom P, Barrett LF (2011). More than a body: Mind perception and the nature of objectification. Journal of Personality and Social Psychology.

[CR35] Harris LT, Fiske ST (2007). Social groups that elicit disgust are differentially processed in mPFC. Social Cognitive and Affective Neuroscience.

[CR36] Haxby JV, Gobbini MI, Calder AJ, Rhodes G, Johnson MH, Haxby JV (2011). Distributed neural systems for face perception. *The Oxford handbook of face perception*.

[CR37] Heflick NA, Goldenberg JL, Cooper DP, Puvia E (2011). From women to objects: Appearance focus, target gender, and perceptions of warmth, morality and competence. Journal of Experimental Social Psychology.

[CR38] Hofree G, Ruvolo P, Bartlett MS, Winkielman P (2014). Bridging the mechanical and the human mind: spontaneous mimicry of a physically present android. PloS one.

[CR39] Holland E, Koval P, Stratemeyer M, Thomson F, Haslam N (2017). Sexual objectification in women’s daily lives: A smartphone ecological momentary assessment study. British Journal of Social Psychology.

[CR40] Hutchings PB, Haddock G (2008). Look Black in anger: The role of implicit prejudice in the categorization and perceived emotional intensity of racially ambiguous faces. Journal of Experimental Social Psychology.

[CR41] Jones BA, Griffiths KM (2015). Self-objectification and depression: An integrative systematic review. Journal of Affective Disorders.

[CR42] Kilbourne, J., & Jhally, S. (2000). Killing us softly 3: Advertising’s image of women [video-recording]. Available from http://www.mediaed.org

[CR43] Koval P, Holland E, Zyphur MJ, Stratemeyer M, Knight JM, Bailen NH, Thompson RJ, Roberts T-A, Haslam N (2019). How does it feel to be treated like an object? Direct and indirect effects of exposure to sexual objectification on women’s emotions in daily life. Journal of Personality and Social Psychology.

[CR44] Loughnan, S., Haslam, N., Murnane, T., Vaes, J., Reynolds, C., & Suitner, C. (2010). Objectification leads to depersonalization: The denial of mind and moral concern to objectified others. *European Journal of Social Psychology*, n/a-n/a. 10.1002/ejsp.755

[CR45] Loughnan, S., Fernandez-Campos, S., Vaes, J., Anjum, G., Aziz, M., Harada, C., ... & Tsuchiya, K. (2015). Exploring the role of culture in sexual objectification: A seven nations study. *Revue internationale de psychologie sociale*, *28*(1), 125–152.

[CR46] Lundqvist, D., Flykt, A., & Öhman, A. (1998). *The Karolinska directed emotional faces*. Stockholm, Sweden: Department of Clinical Neuroscience, Psychology Section, Karolinska Institutet. ISBN 91-630-7164-9.

[CR47] Ma DS, Correll J, Wittenbrink B (2015). The Chicago face database: A free stimulus set of faces and norming data. Behavior Research Methods.

[CR48] Maoz K, Eldar S, Stoddard J, Pine DS, Leibenluft E, Bar-Haim Y (2016). Angry-happy interpretations of ambiguous faces in social anxiety disorder. Psychiatry Research.

[CR49] Mikhailova ES, Vladimirova TV, Iznak AF, Tsusulkovskaya EJ, Sushko NV (1996). Abnormal recognition of facial expression of emotions in depressed patients with major depression disorder and schizotypal personality disorder. Biological Psychiatry.

[CR50] Mitchell RLC, Phillips LH (2015). The overlapping relationship between emotion perception and theory of mind. Neuropsychologia.

[CR51] Murnen, S. K., Smolak, L., Mills, J. A., & Good, L. (2003). Thin, Sexy Women and Strong, : Grade-School Children’s Responses to Objectified Images of Women and Men. *Sex Roles, 11*. 10.1023/A:1025868320206.

[CR52] Nabi RL (2002). The theoretical versus the lay meaning of disgust: Implications for emotion research. Cognition & Emotion.

[CR53] Nowakowski ME, McFarlane T, Cassin S (2013). Alexithymia and eating disorders: A critical review of the literature. Journal of Eating Disorders.

[CR54] Palermo R, Rhodes G (2007). Are you always on my mind? A review of how face perception and attention interact. Neuropsychologia.

[CR55] Peat CM, Muehlenkamp JJ (2011). Self-Objectification, Disordered Eating, and Depression: A Test of Mediational Pathways. Psychology of Women Quarterly.

[CR56] Puvia E, Vaes J (2013). Being a body: Women’s appearance related self-views and their dehumanization of sexually objectified female targets. Sex Roles.

[CR57] Quinn DM, Kallen RW, Twenge JM, Fredrickson BL (2006). The Disruptive Effect of Self-Objectification on Performance. Psychology of Women Quarterly.

[CR58] Rohlinger DA (2002). Eroticizing men: Cultural influences on advertising and male objectification. Sex Roles.

[CR59] Russell JA, Fehr B (1987). Relativity in the perception of emotion in facial expressions. Journal of Experimental Psychology: General.

[CR60] Schmidt AF, Kistemaker LM (2015). The sexualized-body-inversion hypothesis revisited: Valid indicator of sexual objectification or methodological artifact?. Cognition.

[CR61] Steer A, Tiggemann M (2008). The role of self-objectification in women’s sexual functioning. Journal of Social and Clinical Psychology.

[CR62] Susskind JM, Anderson AK (2008). Facial expression form and function. Communicative & Integrative Biology.

[CR63] Swim JK, Hyers LL, Cohen LL, Ferguson MJ (2001). Everyday sexism: Evidence for its incidence, nature, and psychological impact from three daily diary studies. Journal of Social Issues.

[CR64] Tarr M (2013). Perception isn’t so simple. Commentary on ‘Integrating sexual objectification with object versus person recognition: The sexualized-body-inversion hypothesis (Bernard et al., 2012)’. Psychological Science.

[CR65] Teufel C, Alexis DM, Clayton NS, Davis G (2010). Mental-state attribution drives rapid, reflexive gaze following. Attention, Perception, & Psychophysics.

[CR66] Tiggemann M, Williams E (2012). The Role of Self-Objectification in Disordered Eating, Depressed Mood, and Sexual Functioning Among Women: A Comprehensive Test of Objectification Theory. Psychology of Women Quarterly.

[CR67] Troje NF, Bülthoff HH (1996). Face recognition under varying poses: The role of texture and shape. Vision Research.

[CR68] Vaes J, Cogoni C, Calcagnì A (2020). Resolving the Human–Object Divide in Sexual Objectification: How We Settle the Categorization Conflict When Categorizing Objectified and Nonobjectified Human Targets. Social Psychological and Personality Science.

[CR69] Vaes J, Cristoforetti G, Ruzzante D, Cogoni C, Mazza V (2019). Assessing neural responses towards objectified human targets and objects to identify processes of sexual objectification that go beyond the metaphor. Scientific Reports.

[CR70] Vaes J, Paladino P, Puvia E (2011). Are sexualized women complete human beings? Why men and women dehumanize sexually objectified women: Dehumanization of sexually objectified women. European Journal of Social Psychology.

[CR71] Vaillancourt T, Sharma A (2011). Intolerance of sexy peers: Intrasexual competition among women. Aggressive behavior.

[CR72] Wheatley T, Weinberg A, Looser C, Moran T, Hajcak G (2011). Mind Perception: Real but Not Artificial Faces Sustain Neural Activity beyond the N170/VPP. PLoS ONE.

[CR73] Widen SC, Russell JA (2004). The relative power of an emotion’s facial expression, label, and behavioral consequence to evoke preschoolers’ knowledge of its cause. Cognitive Development.

[CR74] Widen SC, Russell JA (2010). Children’s scripts for social emotions: Causes and consequences are more central than are facial expressions. British Journal of Developmental Psychology.

[CR75] Wieser MJ, Brosch T (2012). Faces in context: a review and systematization of contextual influences on affective face processing. Frontiers in psychology.

[CR76] Wilson JP, Young SG, Rule NO, Hugenberg K (2018). Configural processing and social judgments: Face inversion particularly disrupts inferences of human-relevant traits. Journal of Experimental Social Psychology.

[CR77] Wingenbach TSH, Ashwin C, Brosnan M (2018). Sex differences in facial emotion recognition across varying expression intensity levels from videos. PLoS ONE.

[CR78] Wright PJ, Tokunaga RS (2016). Men’s objectifying media consumption, objectification of women, and attitudes supportive of violence against women. Archives of Sexual Behavior.

[CR79] Zogmaister C, Durante F, Mari S, Crippa F, Volpato C (2020). Measuring objectification through the Body Inversion Paradigm: Methodological issues. PLoS ONE.

